# Concomitant protein pathogenesis in Parkinson’s disease and perspective mechanisms

**DOI:** 10.3389/fnagi.2023.1189809

**Published:** 2023-04-27

**Authors:** Yuliang Han, Zhuohao He

**Affiliations:** ^1^Interdisciplinary Research Center on Biology and Chemistry, Shanghai Institute of Organic Chemistry, Chinese Academy of Sciences, Shanghai, China; ^2^University of the Chinese Academy of Sciences, Beijing, China; ^3^Center for Excellence in Brain Science and Intelligence Technology, Chinese Academy of Sciences, Shanghai, China

**Keywords:** comorbidity, pathogenesis, Parkinson’s disease, mechanism, synucleinopathies

## Abstract

Comorbidity is a common phenotype in Parkinson’s disease (PD). Patients with PD not only have motor deficit symptoms, but also have heterogeneous non-motor symptoms, including cognitive impairment and emotional changes, which are the featured symptoms observed in patients with Alzheimer’s disease (AD), frontotemporal dementia (FTD) and cerebrovascular disease. Moreover, autopsy studies have also confirmed the concomitant protein pathogenesis, such as the co-existences of α-synuclein, amyloid-β and tau pathologies in PD and AD patients’ brains. Here, we briefly summarize the recent reports regarding the comorbidity issues in PD from both clinical observations and neuropathological evidences. Furthermore, we provide some discussion about the perspective potential mechanisms underlying such comorbidity phenomenon, with a focus on PD and related neurodegenerative diseases.

## 1. Introduction

Comorbidities are prevalent in many neurodegenerative diseases. The symptoms of patients with certain neurodegenerative disease are frequently mixed with similar symptoms that are featured by another neurodegenerative disease. The pathologies presented in the brains of the patients with neurodegenerative diseases frequently include more than one hallmark proteins (Gorell et al., 1994; Lomen-Hoerth et al., 2002; Bahia et al., 2013; Irwin et al., 2013; Robinson et al., 2021). Moreover, studies have reported the protein–protein interactions among these neuropathological hallmark proteins, suggesting certain mechanisms underlying the pathogenesis of concomitant protein pathogenesis (Giasson et al., 2003; Badiola et al., 2011; Nonaka et al., 2018; Bassil et al., 2020, 2021; Dhakal et al., 2022). Here, we summarize recent data about this comorbidity issue in Parkinson’s disease, from clinical overlapped symptoms, to molecular synergistic interactions, hoping to inspire the efforts to understand the mechanisms and biological implications of the comorbidities in PD and other related neurodegenerative diseases.

## 2. The PD-related comorbidities

### 2.1. PD-related symptomatic comorbidities

#### 2.1.1. PD-like motor symptoms in other diseases

PD is one of the most common neurodegenerative diseases, that is characterized clinically by the motor deficit, and neuropathologically by the neuronal α-synuclein deposits and dopaminergic neuronal loss. PD-related symptoms include tremor, slow movement, rigid muscle and other motor deficit symptoms, which together are usually referred as Parkinsonism. However, the Parkinsonism is also implicated in many other neurodegenerative diseases, such as multiple system atrophy (MSA), amyotrophic lateral sclerosis (ALS), FTD, and AD.

MSA is an atypical movement disorder, and its neurohistological features are the pathological α-synuclein in neuron as Lewy body (LB), and in oligodendrocyte as glial cytoplasmic inclusion (GCI). MSA could be classified into two main subtypes based on the predominant symptoms: MSA predominant Parkinsonism (MSA-P), and MSA predominant cerebellar ataxia (MSA-C). MSA-P is the most common type, and the clinical symptoms of the MSA-P patients are similar to typical PD patients (Wenning et al., 1997), making it difficult to clinically distinguish MSA-P from PD patients.

Motor deficits are also shared symptoms in patients with other neurodegenerative diseases that are not synucleinopathies, such as FTD. The neuropathological term for FTD patients is frontotemporal lobar degeneration (FTLD). According to the predominant pathological protein types, that is either microtubule binding protein tau or TAR DNA-binding protein 43 (TDP-43), it could be grouped into two main subtypes, FTLD-tau and FTLD-TDP. Patients with FTLD-Tau frequently have abnormalities in behaviors and personalities, which are similar to some PD patients (Bahia et al., 2013). FTLD-tau could be further classified into several subtypes, including corticobasal degeneration (CBD), progressive supranuclear palsy (PSP), and Pick’s disease (PiD). CBD is featured by asymmetric motor abnormalities, such as rigidity, bradykinesia and gait disorder (Murray et al., 2007). Patients with PSP variably show postencephalitic parkinsonism, gait disturbance and asymmetric dystonia, hard to be distinguished from PD by clinical symptoms (Williams and Lees, 2009).

Motor deficits are also seen in patients with FTLD-TDP, which is also often referred as FTD with motor neuron disease (MND). According to clinical studies, approximately 15% patients that were diagnosed as FTD met the criteria for MND/FTD (Hodges et al., 2003). MND is a group of disorders clinically featured by weakness, muscular wasting, fasciculations, spasticity, as well as breathing and swallowing problems. Along these symptoms, there are motor neuron loss. ALS is the most common MND pathologically featured by the ubiquitinated cytoplasmic inclusions of TDP-43. Progressive motor neuron loss in ALS patients leads to motor deficit, that is similar to PD and FTD-MND (Lillo and Hodges, 2009). In addition, parkinsonism is also a common secondary symptom in many other diseases, such as cerebrovascular disease (also called vascular parkinsonism), traumatic injury and cerebroma (Stephen and Williamson, 1984; Krauss et al., 1995; Nanhoe-Mahabier et al., 2009; McKee et al., 2012; Tansey et al., 2022).

#### 2.1.2. Non-motor abnormalities in PD

Although classically considered as a movement disorder, PD patients also frequently present various non-motor abnormalities, including cognitive impairment, depression and sleep disorder, which are often regarded as key features in other disease, like AD and FTD. Moreover, these non-motor abnormalities seem correlate with disease progress and patient survival (Irwin et al., 2013).

Cognitive impairment is frequently observed in PD patients, which are recognized as Parkinson’s disease with dementia (PDD). It is reported that almost 24% PD patients have cognitive impairment at the initial diagnosis, and approximately 80% PD patients have cognitive issue over the course of their diseases (Irwin et al., 2013). Thus, cognitive dysfunction is also regarded as a natural consequence at later stage of PD, which shares some similarities as in AD and FTD patients (Bang et al., 2015; Knopman et al., 2021).

Depression is a common symptom in elderly people as well as in many other chronic or disabling diseases. However, it is reported that PD patients have a higher incidence of depression (Nilsson et al., 2002). The incidence of clinically relevant depression symptoms in PD patients is around 35% (Aarsland et al., 2011). Moreover, depression has been proposed to facilitate PD disease progression (Nilsson et al., 2001; Aarsland et al., 2011; Roos et al., 2016; Caraci et al., 2018), that PD patients with depression are reported to have a higher risk for developing cognitive impairment.

Despite usually not being considered as a non-motor symptoms of PD, diabetes, especially the type 2 diabetes mellitus (T2DM) is noteworthy to be associated with PD, as well as AD and ALS (Akter et al., 2011; Santiago and Potashkin, 2013; Kioumourtzoglou et al., 2015). Accumulating epidemiological studies show that PD patients with T2DM have more severe motor symptoms, and the accelerated disease progression (Xu et al., 2011).

Other non-motor complications are also reported in PD patients, including anemia, cancer, sleep disorders, hyposmia and so on (Santiago et al., 2017). It is notable that symptomatic comorbidities are common and implicated in many other neurodegenerative diseases as well, such as AD, FTD and ALS. In this mini-review, we just focus on PD-related comorbidities.

### 2.2. PD-related neuropathological comorbidities

#### 2.2.1. PD-related protein pathologies

PD is pathologically featured by α-synuclein aggregate within neurons, which are often in the form of LB and Lewy neurites (LN) (Goedert et al., 2013). In PD brain, it is believed that α-synuclein pathology spreads from brainstem to limbic and neocortical cortex (Braak et al., 2003). The spread of pathological α-synuclein in cortical regions is more extensive and severe in PDD than in PD patients, which is possibly correlated with the fact that the cognitive impairments are more frequently observed in PDD than in PD patients (Irwin et al., 2013). Neuropathogenesis in PD and PDD is heterogeneous. The frequency of comorbid AD pathologies, such as amyloid-β (Aβ) plaques and neurofibrillary tau tangles, is reported to be about 10% in PD, 35% in PDD and 70% in Dementia with Lewy body (DLB) (Smith et al., 2019). Moreover, the tau pathology in the regions with pathological α-synuclein, such as substantia nigra, thalamus, and medulla, is frequently more than that in the corresponding regions in the cases without α-synuclein pathology. Similarly, in PDD and DLB patients with concomitant AD pathologies, the abundance of Aβ plaques is also positively correlated with α-synuclein pathology in the cortical regions (Bassil et al., 2020).

The presence of TDP-43 positive inclusions is also reported in PD and DLB cases. Almost 30% DLB/AD cases have the concomitant TDP-43 protein pathology (Nakashima-Yasuda et al., 2007). Furthermore, there is a significant positive correlation between TDP-43 lesion and pathological feature in DLB, indicating a synergistic effect of TDP-43 in DLB disease progression. Although TDP-43 pathogenesis is present in DLB cases, most TDP-43 aggregates are not co-localized with α-synuclein in these cases, except the rare colocalization of TDP-43 and α-synuclein in dystrophic neurites (Koga et al., 2018).

MSA is featured by the GCI synuclein pathology enriched in striatonigral and olivopontocerebellar regions (Goedert et al., 2013). In some rare cases, TDP-43 aggregates are reported to be present in the dystrophic neurites and perivascular in the medial temporal lobe and subcortical regions of the MSA brains. It is also reported that the colocalizations between pathological α-synuclein and TDP-43 are detected inside the GCIs (Koga et al., 2018).

#### 2.2.2. Synuclein pathology in AD

The neuropathological hallmarks of AD are the accumulation of pathological Aβ plaques and tau tangles in the brain. However, the presence of α-synuclein or/and TDP-43 in AD have also been well observed. The prevalence of aggregated α-synuclein in AD brains is more than 50% (Robinson et al., 2021). Besides the stereotypical spread pattern from brainstem to neocortex, the presence of LB pathology is also reported in the medial temporal lobe, where typical AD pathologies are (Montine et al., 2012). Such differential distributions of LB and AD pathologies indicate neocortical and amygdala predominant LBs might be an AD pathology induced α-synuclein pathology in disease progression rather than an age-related incidental pathology.

#### 2.2.3. Synuclein pathology in FTLD/CTE

The common neuropathological feature of FTLD is the degeneration of frontal or temporal lobes. Based on protein deposits, and regional and cellular distribution patterns, FTLD is classified into FTLD-Tau, FTLD-TDP and the fused in sarcoma protein (FUS) in FTLD, FTLD-FUS (Bahia et al., 2013). FTLD-Tau and FTLD-TDP, respectively, represents around 45% of total FTLD cases, and FTLD-FUS accounts about 5% FTLD cases. Study has reported the existence of α-synuclein pathology in the granule cells of the dentate gyrus and the CA1/Subiculum region of FTLD-Tau cases (Rohan et al., 2015).

Chronic traumatic encephalopathy (CTE) is another tauopathy disease, initially described in the case of dementia pugilistica boxers with neurofibrillary tangles in neocortical areas (Corsellis et al., 1973). Parkinsonism is frequently observed in CTE patients, and the autopsy studies report neuronal loss in the substantia nigra in their brains. CTE cases are heterogeneous, in addition to tau, 37% CTE patients is found to have other abnormally aggregated proteins, including TDP-43, Aβ and α-synuclein, among which, 22% patients developed LB pathologies (McKee et al., 2012).

#### 2.2.4. Synuclein pathology in TDP-43 proteinopathy

TDP-43 proteinopathy is a group of disease featured by TDP-43 pathogenesis, such as ALS and FTLD-TDP, although TDP-43 pathogenesis is also observed in AD and CTE. Several cases of ALS and parkinsonism-dementia complex (ALS/PDC) reported in the Kii Peninsula in Japan showed the phosphorylated α-synuclein in the brainstem and limbic system, with the amygdala as the most severely affected region. Furthermore, the co-existence of TDP-43 inclusion with the neurofibrillary tau tangle, or LB in the same neuron have been reported in these ALS/PDC cases (Kuzuhara, 2007). In addition to ALS, the colocalization between TDP-43 and α-synuclein also have been reported in DLB patients (Higashi et al., 2007).

The Synergistic Interactions between synuclein pathology and other concomitant protein pathologies.

## 3. The Synergistic Interactions between synuclein pathology and other concomitant protein pathologies

### 3.1. **α**-synuclein and A**β**

In addition to the hallmarks Aβ and tau, approximately 50% AD cases also exhibit α-synuclein pathology (Robinson et al., 2021). Meanwhile, in addition to featured α-synuclein pathology, Aβ pathology is also detected in DLB cases (Kantarci et al., 2020). The prevalence of the concomitant pathological Aβ and α-synuclein in AD and DLB cases suggests there are synergistic pathogenesis effects between these two proteins in different diseases. *In vitro* studies have shown that α-synuclein and Aβ could form hetero-oligomers and facilitate the aggregation of each other. *In vivo* studies also have shown the present of Aβ pathology facilitates α-synuclein transmission and toxicity (Masliah et al., 2001; Tsigelny et al., 2008; Ono et al., 2012; Chia et al., 2017; Bassil et al., 2020).

Conversely, α-synuclein also exerts effect on Aβ pathogenesis. Introducing transgenic mutant α-synuclein into the 3xTransgenic (Tg) AD mouse model, which contains three mutations associated with familial AD (APP Swedish, MAPT P301L, and PSEN1 M146V) and mainly shows progressive Aβ pathogenesis, accelerated Aβ pathology (Clinton et al., 2010). On the other hand, reducing α-synuclein expression in an AD mouse model reversed the associated neurodegenerative phenotype. In APP Tg mice, knocking out endogenous α-synuclein prevented the degeneration of cholinergic neurons and improved the behavioral deficits of APP Tg mice, although the changes in APP gene expression or the Aβ plaque burden were not detected (Spencer et al., 2016). Together, these results indicate that α-synuclein participates in Aβ pathogenesis, and possibly causes cell vulnerability of specific neuronal population in AD.

### 3.2. **α**-synuclein and tau

*In vitro* studies have shown α-synuclein and tau have synergistic fibrilization effect on each other (Giasson et al., 2003; Badiola et al., 2011; Guo et al., 2013; Pan et al., 2022). Moreover, α-synuclein has been also shown to induce tau aggregation in a process termed cross-seeding (Giasson et al., 2003; Guo et al., 2013; Bassil et al., 2020). *In vivo* studies in DLB and AD animal models have indicated a synergistic relationship between α-synuclein and tau (Masliah et al., 2001; Frasier et al., 2005; Höglinger et al., 2005; Duka et al., 2009; Clinton et al., 2010; Khandelwal et al., 2010; Emmer et al., 2011; Haggerty et al., 2011; Morris et al., 2011; Wills et al., 2011; Masuda-Suzukake et al., 2014). Double transgenic mice with both tau and α-synuclein transgenes exhibited increased pathologies of each other compared to the mice with only one transgene (Masliah et al., 2001; Clinton et al., 2010). However, the interactions between α-synuclein and tau seem not so straight forward. In Tg mouse models, reducing the expression of α-synuclein in a tau transgenic mouse or reducing tau expression in an α-synuclein transgenic mouse did not decrease pathogenesis of the other protein (Morris et al., 2011). In seeding mouse models, seeding human derived pathological tau into WT or Aβ biogenic mouse model, that could induce pathological tau comprised of endogenous murine tau, failed to induce α-synuclein pathogenesis simultaneously (He et al., 2018). However, in another experiment, the co-injection of the mixture of α-synuclein PFFs and human brain-derived pathological tau induced more tau pathology than the injection of human brain-derived pathological tau alone in the WT mice. Moreover, knocking out α-synuclein decreased the induced tau pathology burden in non-tau Tg mice by inoculation of human brain-derived pathological tau. Interestingly, such synergistic effect seems not bidirectional, as the level of α-synuclein pathology remains unaffected when the α-synuclein PFFs were injected into the WT mice with or without the induction of endogenous tau pathogenesis by human brain-derived pathological tau. Similarly, knocking out tau did not affect the induced synuclein pathology by inoculation of α-synuclein PFF (Bassil et al., 2020). Thus, α-synuclein is considered as a modulator in tau pathogenesis and spreading, whereas tau might play much less similar roles in modulating α-synuclein pathogenesis and spreading.

### 3.3. **α**-synuclein and TDP-43

The synergistic interactions between α-synuclein and TDP-43 have also been reported in terms of their fibril assembly and cellular cytotoxicity. Notably, although either soluble or pathological α-synuclein promoted the fibrillization and liquid droplets formation of the C-terminal domain of TDP-43 (TDP-43 PrLD), TDP-43 PrLD fibrils failed to templating α-synuclein in *in vitro* experiments, indicating a non-bidirectional modulation on the pathogenesis between α-synuclein and TDP43, similar to the interaction between α-synuclein and tau as summarized above (Dhakal et al., 2021, 2022). However, *in vivo* evidence supports a mutual synergistic interaction between them. More α-synuclein pathogenesis and neurodegeneration was detected in the *C. elegans* with the co-expression of transgenic TDP-43 and α-synuclein than in the worms that only had α-synuclein transgenic expression (Shen et al., 1866). Furthermore, double transgenic mice with both mutant human TDP-43 and mutant α-synuclein showed enhanced dopaminergic neuronal degeneration compared to the transgenic mice that only overexpress α-synuclein (Tian et al., 2011).

Conversely, it is reported that α-synuclein fibrils promote the phosphorylation and aggregation of TDP-43 protein. In SH-SY5Y cells expressing nuclear localization signal (TDP dNLS)-deleted TDP-43 truncation and α-synuclein, preformed synthetic α-synuclein fibrils were sufficient to induce abundant aggregates of phosphorylated TDP-43, which were partially co-localized with phosphorylated α-synuclein. *In vivo* study also showed the presence of phosphorylated TDP-43 in wild type mouse brains 1 month after the inoculation of preformed mouse α-synuclein fibrils, which were also rarely co-localized with α-synuclein pathology (Masuda-Suzukake et al., 2014).

## 4. Perspective mechanisms underlying co-pathologies

### 4.1. Potential mechanisms underlying synucleinopathies

α-synuclein pathology is present in almost all the PD patients, including sporadic and familial patients, and the pathological burden correlates with clinical symptoms (Poulopoulos et al., 2012), indicating its key roles in dopamine (DA) neuron degeneration and parkinsonism phenotype. In genome-wide association studies (GWAS) analysis, the mutations and overexpression of α-synuclein encoding gene SNCA have been reported in both familial and sporadic PD patients (Singleton et al., 2003; Simon-Sanchez et al., 2009), indicating a causal link between synuclein pathology and PD. The disease-associated mutants and misfolding have been shown sufficient to induce synuclein pathogenesis (Dawson et al., 2010; Luk et al., 2012). Mechanisms have been proposed to for such process, including the initial formation, subsequent spread and the clearance of aggregated α-synuclein. Genetic mutation in SNCA is one of the most accepted mechanisms that account for initial α-synuclein pathogenesis. SNCA Glu46Lys, Ala53Thr are two well-known pathogenic mutations identified in early familial PD patients. *In vitro* assays showed such mutations could accelerate α-synuclein aggregation (Giasson et al., 2002; Emmer et al., 2011). Transgenic mice, respectively, overexpressing each of these mutant synuclein developed abundant α-synuclein pathology *in vivo* (Rochet et al., 2004; Forman et al., 2005; Savitt et al., 2006). Besides the disease causal SNCA mutations, there are other genetic risk factors for PD, including E3 ubiquitin-protein ligase parkin (*parkin*), Serine/threonine-protein kinase PINK1 (*PINK1*), Parkinson disease protein 7 (*DJ-1*), Leucine-rich repeat serine/threonine-protein kinase 2 (*LRRK2*) and glucocerebrosidase (*GBA*).

*Parkin* is associated with protein clearance pathways, and is reported to co-localize with α-synuclein within the LB inclusions. *Parkin* mutations are involved in the aggregation of misfolded α-synuclein within neurons in the substantia nigra pars compacta (SNpc) (Schlossmacher et al., 2002). Moreover, *Parkin* mutations are reported to significantly decrease the proteasomal degradation process by regulating ubiquitin-ligase enzymatic activities (Shimura et al., 2000, 2001), which are involved in the process of α-synuclein pathogenesis.

*GBA* encodes the lysosomal enzyme glucocerebrosidase (GCase). Mutations in *GBA* are linked to PD and DLB. The *GBA* mutants are reported to cause alterations in lipid levels and are associate with α-synuclein pathogenesis (Velayati et al., 2010). Nearly all patients that carried *GBA* mutations eventually developed α-synuclein pathology. Importantly, GCase loss of activity impairs the autophagy lysosomal pathway, and GCase deficiency enhanced spreading of α-synuclein pathology (Gegg et al., 2020; Henderson et al., 2020).

*DJ-1* has been shown to be involved in many signaling pathways, including cell survival (PI3K/Akt pathway), transcriptional regulation, anti-oxidation and protein degradation pathways. *DJ-1* can bind to several chaperones, including HSP70, carboxy-terminus of HSP70-interacting protein (CHIP), and mitochondrial HSP70/mortalin/Grp75. The co-localization of *DJ-1* and α-synuclein indicates a potential role of *DJ-1* in α-synuclein pathogenesis, although their clear connections remain to be elusive (Cardinale et al., 2014; Blaszczyk, 2016).

*PINK1* is a serine/threonine-protein kinase which is reported to exert a potential neuronal protective function in response to stress-induced mitochondrial damage (Wu et al., 2015). Mutations in *PINK1* gene are linked to mitochondrial dysfunctions and the SNpc neuronal degeneration, which is directly associated with the development of PD symptoms. However, the neuropathological data from autopsies of patients carrying *PINK1* mutations are very limited, and it is unclear whether *PINK1* is associated with α-synuclein pathogenesis in patients.

*LRRK2* is another common genetic risk of PD, although the effects of different *LRRK2* mutations on PD pathogenesis are inconclusive. It has been reported that *LRRK2* activity does not alter seeded α-synuclein pathologenesis in primary neurons and non-transgenic mice (Henderson et al., 2018, 2019). Moreover, a subset of patients with *LRRK2* mutations showed the absence of LB pathology at autopsy, not suggesting a direct relationship between *LRRK2* and the synuclein pathogenesis (O'Hara et al., 2020).

Besides genetic factors, cell biological processes are also proposed to play important roles in PD pathogenesis, including mitochondrial damage, oxidative stress, neuroinflammation, lipid metabolism and protein degradation (Goedert et al., 2013). Mitochondrial impairment and oxidative stress are believed to be involved in PD-related dopaminergic neuronal loss. It is reported that the transplantation of PD patient-derived mitochondrial DNA (mtDNA) into neuroblastoma cells lead to the detection of LB-like pathology in the cells (Wilkins et al., 2014).

Ubiquitin-Proteasome System (UPS) is one of the protein degradation pathways that are responsible for eliminating misfolded proteins (Tan et al., 2008; Deleidi and Maetzler, 2012; Tanik et al., 2013; Liu et al., 2016; Yu et al., 2019; Bassil et al., 2020). Failures of this critical cellular system have been observed in PD, which could account for the aggregation of misfolded amyloid proteins. The PD risk genes, such as *Parkin* and *UCH-L1*, along with UPS, are all involved in the degradation of misfolded α-synuclein. Inhibition of proteasome system by Lactacystin resulted in LB-like deposits in the fetal rat ventral mesencephalic cells (McNaught et al., 2002; Rideout et al., 2005). Low level proteasome inhibition by MG115 in human neuroblastoma cells (SH-SY5Y) for several weeks resulted in mitochondrial damage, elevated protein oxidation and aggregates (Ding et al., 2003; Sullivan et al., 2004).

Molecular chaperones have also been shown to bind to α-synuclein oligomers or pre-fibrillar structures, and are supposed to reduce their toxicity. In particular, the overexpression of chaperone heat shock protein 110 (HSP110) in α-synuclein transgenic mice prevented the formation of α-synuclein aggregation in the brain (Taguchi et al., 2019). In addition, HSPs also play pivotal roles in the function of ubiquitin proteasome and the autophagy-lysosomal pathways. Large protein debris, such as fibrils of α-synuclein, could not be efficiently degraded through UPS. Chaperone-mediated autophagy (CMA) executes protein degradation with the aid of heat-shock cognate protein (HSC70), which specifically bind to proteins to be degraded via specific pentapeptide targeting motif (KFERQ). Experimental evidence showed that down-regulation of autophagy-related genes, Atg5 or Atg7, in the CNS lead to the aggregation of poly-ubiquitinated protein debris in neurodegenerative disease animal models (Komatsu et al., 2006). Thus, the CMA deficit could decrease the efficiency of degrading large protein aggregates, such as pathological α-synuclein, causing excess accumulation of protein aggregates in neural cells.

In addition, low pH is reported to increase fibril fragmentation, mimic the scenario of the synuclein pathogenesis inside the endosomes and lysosomes, where the environment is acidic (Buell et al., 2014). Many posttranslational modification are also reported to promote *in vitro* fibrillation of α-synuclein, such as N-terminal and C-terminal truncation (Terada et al., 2018). However, the Ser129 phosphorylation on synuclein monomer is reported to inhibit the synuclein fibrillation in the *in vitro* experimental system (Ghanem et al., 2022). Other factors affecting α-synuclein fibrillation include certain glycosaminoglycans, such as heparin; metals, such as aluminum, copper and iron; and oxidative/nitrative challenge, such as hydrogen peroxide and peroxynitrite treatment. These co-factors or microenvironmental changes could facilitate the conformational transformation of α-synuclein, through the changes in electrostatic interactions between synuclein and other environment factors, or among α-synuclein monomers (Fink, 2006).

### 4.2. Perspective mechanisms underlying synucleinopathy-related co-pathogenesis

Although PD is a featured synucleinopathy, the concomitant protein pathogenesis is prevalent in PD-related neurodegenerative diseases. Here we provide a perspective discussion about the potential mechanisms underlying the presence of synuclein-related co-pathogenesis.

#### 4.2.1. Defective global protein degradation

The postmitotic status of neuron increases their vulnerability to malfunction in proteome homeostasis (proteostasis). The deficiencies of protein degradation systems are frequently reported in neural cells in neurodegenerative disease patients and animal models. Alternations in the autophagy-lysosomal pathway, UPS, and CMA have been reported along with the presence of αsynuclein, tau, Aβ pathologies (Cuervo et al., 2004; Bassil et al., 2020; Bourdenx et al., 2021). CMA and UPS play primary roles in degrading soluble proteins, and macroautophagy participates in sequestering and clearing larger protein inclusions (Bourdenx et al., 2021). In the cases with co-pathologies of both αsynuclein and tau, UPS is altered, as indicated by the increase of UPS markers, including ubiquitin, K48 and K63 polyubiquitin chains. Moreover, autophagy machinery is also impaired in the present of such co-pathologies, as indicated by the increase in LC3-II and P62 (Bassil et al., 2020).

**Figure 1:**
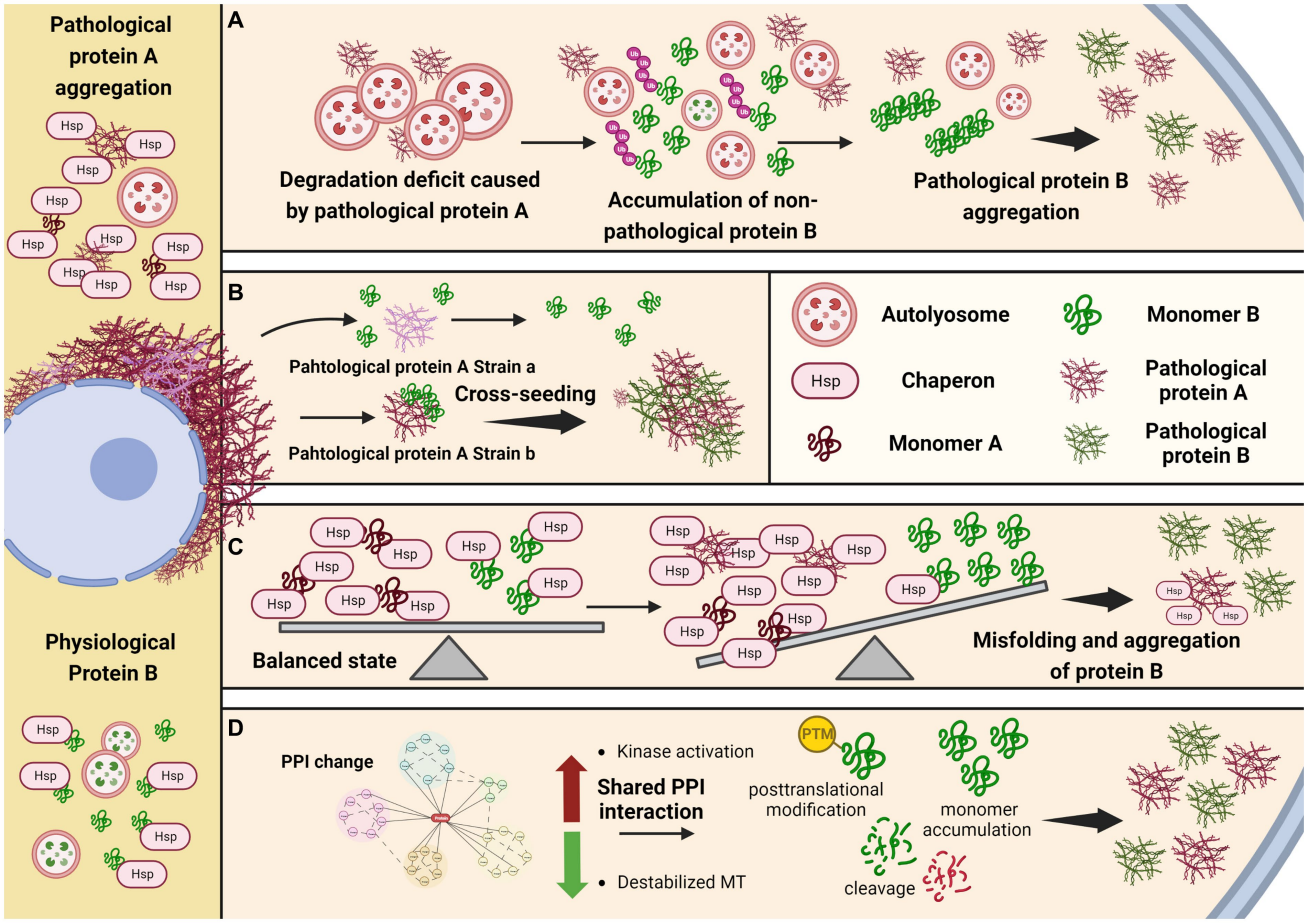
Potential mechanisms underlying comorbid pathogenesis in neurodegenerative diseases. **(A)** Protein aggregation caused by defective global protein degradation. **(B)** Specific conformational strain cross-seeding the other amyloid protein. **(C)** Loss of protein quality control due to limited chaperone molecule. **(D)** Shared regulation pathway and posttranslational modification facilitate the aggregation of the other protein.

In addition to autophagy-lysosomal pathway, the CMA or UPS pathways may be also involved in the pathogenesis in another way, that the presence of one pathological protein could compromise the degradation of the physiological form of the other protein, by consuming extra CMA or UPS resources, resulting in the abnormal enrichment of the other protein. Certain protein, such as tau, at higher concentration is prone to adopt abnormal conformation and proceed into misfolding assembly and pathological transformation (Santacruz et al., 2005; Adams et al., 2009; DeVos et al., 2017). Thus, global protein degradation impairments could be involved in the co-pathogenesis process (Figure 1A).

#### 4.2.2. Cross-seeding and co-deposition

The hallmark proteins in many neurodegenerative diseases are intrinsic disorder protein without well-defined secondary or tertiary structures under physiological conditions, but could acquire specific pathogenic conformations and exert distinct toxicities under pathological conditions. Studies from *in vitro* fibrilization reactions, culture cells, and *in vivo* mouse models have demonstrated the prion-like propagation of these pathogenic protein, that the protein with misfolded conformations could be amplified without nuclear acid materials. As long as the protein acquired the disease-associated conformation, it could function as the seed to template and transform the same protein to change from physiological status into pathological status. Moreover, it has been reported that many pathological proteins in different neurodegenerative diseases share the common β-pleated sheet conformation, which provides the capacity to cross-seed other protein that have the potential to undergo pathological transformation (Giasson et al., 2003; Mandal et al., 2006; Tsigelny et al., 2008; Ono et al., 2012; Guo et al., 2013). It is noticeable that, although pathological strains could be assembled by the same monomer protein, the seeding abilities of these strains are different, indicating the cross-seeding ability is correlated with specific conformation or other co-factors, in addition to the shared β-pleated sheet conformation (Guo et al., 2013). Structural studies of the pathological protein fibrils derived from patients’ postmortem tissues showed the presence of different pathological strains in these diseases (Strohaker et al., 2019; Lovestam et al., 2022; Yang et al., 2022), supporting that fibril structure determines its seeding and pathogenic ability (Figure 1B).

#### 4.2.3. Limited chaperone function

Molecular chaperone is an important element in controlling the protein quality (Hartl et al., 2011). Several proteins have been reported to, respectively, bind and exert chaperone functions in the aggregation of α-synuclein, tau, TDP-43 and FUS amyloids (Burmann et al., 2020; Li et al., 2022; Lu et al., 2022; Zhang et al., 2022). For example, Hsp27 is reported to effectively reduce the aggregation of tau *in vitro* (Baughman et al., 2018), and rescue tau pathology in transgenic drosophila and mouse models (Abisambra et al., 2010; Zhang et al., 2022). It is possible that those molecular chaperones could bind and stabilize different proteins, but the chaperon functions are variable to different proteins, leading to the co-pathogenesis. In PD and DLB, the co-pathogenesis of α-synuclein and tau is possible due to differential effects on each of them by the same chaperon. Such chaperone might bind to both α-synuclein and tau protein, with different affinities. Moreover, it’s binding affinity for α-synuclein or tau differs between their soluble and insoluble phases, with higher binding affinity to the aggregated form. When α-synuclein undergoes pathogenesis, more chaperone would be recruited to bind such pathological α-synuclein in order to prevent its misfolding and aggregation. On the other hand, the same chaperone that originally also bind to and stabilize tau, although with a lower affinity, would be reduced due to limited total amount, leading to the vulnerability of tau pathogenesis (Figure 1C). 14-3-3 might be one of such chaperons, as it shares a homology region with α-synuclein, and is also shown to potentially bind tau (Ostrerova et al., 1999; Chen et al., 2019).

#### 4.2.4. Shared regulatory pathways and posttranslational modifications

Shared protein–protein interaction (PPI) could be another potential mechanism underlying co-pathology. It has been shown that tubulin could be destabilized by αsynuclein, resulting in the increase of unbound and destabilized tau, making it prone to form tau aggregation (Esposito et al., 2007; Moussaud et al., 2014). Thus, the destabilization of microtubules might contribute to the tau pathogenesis in the synucleinopathies.

Besides, kinase-dependent mechanisms might also mediate tau aggregation in synucleinopathies. Tau has been found to be hyperphosphorylated in αsynuclein transgenic mice, in which the activities of ERK and JNK, which are kinases able to phosphorylate tau, are increased (Frasier et al., 2005; Kaul et al., 2011; Oaks et al., 2013). *In vitro* studies also reported another two tau kinases GSK3β and protein kinase A (PKA) could be activated by αsynuclein, and promote tau phosphorylation at their specific phosphorylation sites (Jensen et al., 1999; Duka et al., 2006, 2009).

Moreover, recent studies reported an asparagine endopeptidase (AEP) with broad cleavage substrates, including APP, tau, α-synuclein and TDP-43. And the truncated products have higher tendency to form aggregation (Zhang et al., 2016). Thus, the activation of AEP and upstream C/EBPβ/δ-secretase pathway may also be involved in the copathogenesis of αsynuclein and tau (Figure 1D).

## 5. Summary

Accumulating attentions have been raised on the concomitant protein pathogenesis issues in recent neurodegenerative disease studies. Patients with PD and related DLB are frequently reported to have overlapped syndromes and neuropathologies with other neurodegenerative diseases. Experimental studies have demonstrated the synergistic interactions between α-synuclein and other neuropathologic proteins. Simultaneous development of different pathologies accelerates the disease progression. Although the treatment of levodopa and deep brain stimulation (DBS) could improve the clinical symptom of PD, the intervention of the α-synuclein pathogenesis progress inside the patients’ brains is still very limited, which eventually would make the patients’ conditions worsen. Currently, many clinical trials for such neurodegenerative diseases are targeting single pathological protein. However, the outcomes are not very encouraging yet. Thus, it needs to consider new strategies with targeting multiple concomitant pathological proteins in treating such devastating diseases. Targeting different concomitant protein pathogenesis could hopefully decrease their synergistic interaction to each other, delay the disease progression, and relieve the patients’ symptoms. However, the mechanisms underlying the co-pathogenesis in different neurodegenerative diseases remain largely unclear, and need to be addressed before we could develop more effective therapeutic approaches.

In this mini-review, we briefly summarize the concomitant protein pathogenesis phenotypes in PD and related diseases. Current studies have reported the complicated interactions between synuclein pathology and other concomitant protein pathologies. However, the underlying mechanisms of the comorbid pathogenesis remain to be further studied. Thus, we propose several perspective mechanisms based on related studies. We suggest that maintaining cellular protein homeostasis; inhibiting overactivated kinases that are involved in disease pathogenesis; normalizing cellular environment, like stabilizing microtubule, could be potential therapeutic interventions of PD and related neurodegenerative diseases.

## Author contributions

YH wrote the initial draft. ZH revised the manuscript. All authors contributed to the article and approved the submitted version.

## Funding

This work was supported by the National Natural Science Foundation of China grant 82171413 (ZH), Science and Technology Commission of Shanghai Municipality (STCSM) grant 21ZR1482900 (ZH) and 2019SHZDZX02 (ZH).

## Conflict of interest

The authors declare that the research was conducted in the absence of any commercial or financial relationships that could be construed as a potential conflict of interest.

## Publisher’s note

All claims expressed in this article are solely those of the authors and do not necessarily represent those of their affiliated organizations, or those of the publisher, the editors and the reviewers. Any product that may be evaluated in this article, or claim that may be made by its manufacturer, is not guaranteed or endorsed by the publisher.
